# Iron Metabolism and Ferroptosis in Peripheral Nerve Injury

**DOI:** 10.1155/2022/5918218

**Published:** 2022-12-02

**Authors:** Lei Huang, Mengxuan Bian, Jian Zhang, Libo Jiang

**Affiliations:** Department of Orthopedic Surgery, Zhongshan Hospital, Fudan University, Shanghai 200032, China

## Abstract

Peripheral nerve injury (PNI) is a major clinical problem that may lead to different levels of sensory and motor dysfunction including paralysis. Due to the high disability rate and unsatisfactory prognosis, the exploration and revealment of the mechanisms involved in the PNI are urgently required. Ferroptosis, a recently identified novel form of cell death, is an iron-dependent process. It is a unique modality of cell death, closely associated with iron concentrations, generation of reactive oxygen species, and accumulation of the lipid reactive oxygen species. These processes are regulated by multiple cellular metabolic pathways, including iron overloading, lipid peroxidation, and the glutathione/glutathione peroxidase 4 pathway. Furthermore, ferroptosis is accompanied by morphological changes in the mitochondria, such as increased membrane density and shrunken mitochondria; this association between ferroptosis and mitochondrial damage has been detected in various diseases, including spinal cord injury and PNI. The inhibition of ferroptosis can promote the repair of damaged peripheral nerves, reduce mitochondrial damage, and promote the recovery of neurological function. In this review, we intend to discuss the detailed mechanisms of ferroptosis and summarize the current researches on ferroptosis with respect to nerve injury. This review also aims at providing new insights on targeting ferroptosis for PNI treatment.

## 1. Introduction

Peripheral nerve injury (PNI) is one of the most commonly reported injury diseases in clinical practice, which represents a significant functional impairment disability [1] and often results in clinical as well as public health problems. It is associated with various causes, including trauma, fracture, traffic accidents, falls, crush injuries, and complications after surgery, and its incidence has been on the rise in recent years [2, 3]. In addition, PNI can lead to a variable extent of autonomic dysfunction, sensory disturbance, partial paralysis, and chronic neuropathic pain (NP), thereby degrading the quality of life [4]. Despite advances in surgical technology, the clinical applications are still limited, and there is an urgent need to further elucidate the mechanism of PNI to develop successful treatment strategies [5]. An increasing number of studies have investigated the mechanisms of nerve injury and confirmed the involvement of cell death with different forms in PNI, such as apoptosis [6], autophagy [7], and necroptosis [8]; however, the major pathway and its molecular regulation in PNI is still undefined. Hence, there may be other forms of cell death that contribute to the imbalanced microenvironment in PNI.

In the nervous system, iron is involved in many important processes, such as oxidative phosphorylation, myelination, and the synthesis as well as metabolism of neurotransmitters [9]. Therefore, iron metabolism needs to be strictly regulated in the human body. Abnormal iron metabolism is closely associated with the defective repair process after a damage to the nervous system, especially in cases of neurodegenerative diseases [10], stroke [11], spinal cord injury (SCI) [12], and PNI [13]. Incidentally, ferroptosis, first defined in 2012, is a new form of regulated cell death (RCD), which is dependent on iron ions, and is driven by lethal lipid peroxidation as well as the lipid reactive oxygen species (ROS) [14]. Therefore, ferroptosis can be suppressed by directly blocking the lipid peroxidation pathways or by depleting the iron reserves via pharmacological means [15]. The biological characteristics of ferroptosis include accumulation of ROS, iron overload, rupture of the outer mitochondrial membrane, and a lack of chromatin condensation [16–18]. Although ferroptosis has been studied extensively in recent years, the mechanism of ferroptosis occurring during PNI remains unclear. In this article, we review the connection between iron metabolism and PNI, thereby attempting to provide novel ideas regarding the potential association of ferroptosis with PNI.

## 2. Peripheral Nerve Tissue

In peripheral nerve system (PNS), peripheral nerves include 12 pairs of cranial nerves and 31 pairs of spinal nerves, mainly composed of neuronal axons and Schwann's cells (SCs), which are glial cells surrounding the axons of peripheral nerves and supporting, nourishing, and protecting the nerves [19]. SCs are wrapped around nerve fibers, which called myelinated nerve fibers, and there are differences in the morphology and function of SCs between myelinated and unmyelinated nerve fibers [20]. The nerve impulse conduction of myelinated nerve fibers jumps from one node of Ranvier to the next in the way of jumping conduction. As the unmyelinated nerve fibers conduct in the form of local current, the conduction speed of myelinated nerve fibers is faster than that of unmyelinated nerve fibers [20, 21]. The myelin sheath is the tubular outer membrane surrounding the axons of the nerve fibers, which is part of the SCs membrane [22]. The cytoplasm of SCs is not only found at the edge and both ends of cells but also in the incisures of Schmidt-Lanterman, which constitutes a spiral cytoplasmic channel communicating with the cytoplasm at the inside and outside of the cells [23]. More importantly, SCs can regulate the axonal activity of presynaptic nerve terminals and produce neurotrophic factors to promote axonal growth. In addition, SCs can also remove axonal myelin debris, secrete a large number of cytokines to act with mast cells, regulate macrophage recruitment, accelerate the degeneration process, and therefore play an important role in the regeneration of injured peripheral nerves [24].

## 3. Pathogenesis of Peripheral Nerve Injury, Neuropathic Pain, and Nerve Regeneration

The maintenance of normal PNS function depends on the integrity of the neural structure. PNI is often described using the Seddon classification [25] and the Sunderland classification [26], which describe three and five degrees of nerve injury, respectively, and further allow a better prediction of outcomes following injury. Neurapraxia is often the result of a compressive or crush injury to the nerve where conduction is blocked due to myelin damage. This leads to a focal conduction block without the Wallerian degeneration as the axon itself remains intact, with recovery generally evident in three to six weeks when myelin continuity is restored [27]. Neurotmesis is the complete physiologic transection of axons as well as all supporting tissue. Neurotmesis results in changes within the nerve cell body and degeneration with a variable recovery course [28]. After the two most severe peripheral nerve injuries, transection and crush injuries, the nerve fibers distal to the site of the injury lose their contact with the neuronal cell body. They are deprived, for all intents and purposes, of their source for synthesis of proteins, lipids, and carbohydrates [29]. Then, lipid metabolism disorder, oxidative stress injury, and iron metabolism disorder in the intracellular environment lead to different kinds of cell death, such as apoptosis or ferroptosis, which further leads to nerve tissue damage and secondary nerve injury [30].

After PNI, a series of reactions occur at the injury site, particularly the retrograde degeneration of the proximal nerve [31]. In this case, the axon and myelin sheath break apart and disintegrate from the proximal stump to the cell, and it is mainly manifested as the enlargement of the neurons, the eccentric shift of the nucleus, and the dissolution or disappearance of the Nissl's bodies. Generally, this process ends in the first collateral branch adjacent to the stump; however, by then, the Wallerian degeneration sets in, i.e., the axons and myelin sheaths of the distal nerves become degenerated [32, 33]. Wallerian degeneration is antegrade degeneration in which the part of the axon separated from the neuron's cell body degenerates distal to the injury [34]. Affected SCs throughout the distal segment begin to break down myelin. This distal degeneration, in conjunction with the aggregation of macrophages to this area, leads to the phagocytosis of axonal and myelin debris. These events eventually lead to the collapse of the myelin canal [27]. Additionally, damaged neurons as well as various cells in the distal nerve stump are involved in the regeneration of peripheral nerves. While damaged neurons switch to the growth mode from their action potential transduction mode, the SCs, macrophages, and endoneurium fibroblasts of the distal nerve stump undergo significant phenotypic changes, thereby creating a growth permissive environment for axon regeneration [35, 36].

PNI could cause disability and NP, which constitutes a substantial economic burden to patients as well as to society. NP is not a single disease but a syndrome caused by several different diseases or lesions, among which PNI is one of the most common diseases [37]. This peripheral sensitization is caused by neuroimmune interactions involving migration and infiltration of immune cells to the site of injury and the release of proinflammatory factors in nerve injury-induced NP. Most importantly, NP is closely associated with nerve regeneration [38] and related with decreased inhibitory neurons or pathological changes in astrocyte activation [39]. SCs are involved in the occurrence and development of nerve injury-induced NP and support peripheral nerve function, playing an important role in the pathological processes of NP. It has been reported that appropriate regulation of SCs autophagy could remove damaged organelles and abnormal proteins, which may be a promising therapeutic strategy for NP [37].

## 4. Iron Metabolism, Characteristics of Ferroptosis, and the Potential Relationship between Ferroptosis and Peripheral Nerve Injury

**Figure 1:**
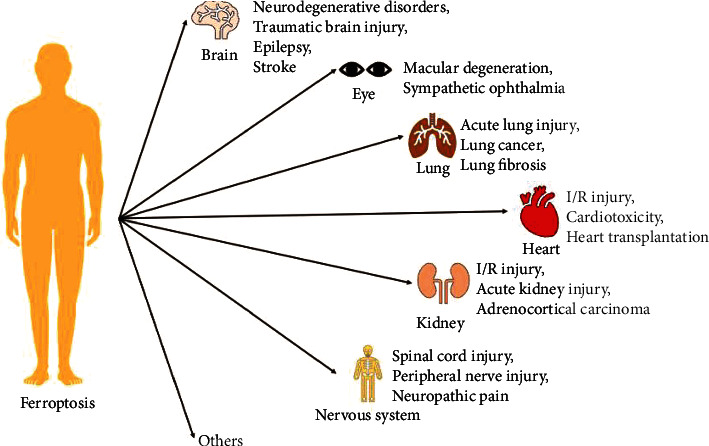
Ferroptosis plays an important role in the development of multiple diseases associated with different systems of the human body.

In the human body, transferrin (TF) transports free iron, which includes ferrous (Fe^2+^) and ferric (Fe^3+^) forms, to various tissues and cells including neurons and SCs. Upon reaching the tissues or cells, the TF-Fe^3+^ complex enters by binding to the membrane protein, transferrin receptor 1 (TFR1). Subsequently, Fe^3+^ is released from TF and reduced to Fe^2+^ under the action of the six-transmembrane protein of prostate 3 (STEAP3) [40]. Free iron is active after dissociation from TF, and the excess intracellular iron is exported through ferroportin (FPN) or stored in the form of Fe^3+^ in ferritin. Iron is also released from endosomes into the labile iron pool (LIP) by divalent metal transporter 1 (DMT1) to avoid cytotoxicity [41]. This is an important physiological process in iron metabolism. When Fe^2+^ levels continuously increase, the Fenton reaction is triggered, in which excess Fe^2+^ reacts with H_2_O_2_ to produce a large amount of hydroxyl radicals; these, in turn, can further aggravate the degree of lipid peroxidation. Additionally, Fe^2+^ also forms an important part of the catalytic subunit of lipoxygenase (LOX), which catalyzes the lipid peroxidation reaction [42]. Therefore, abnormal accumulation of iron can cause adverse effects with respect to neural diseases as well as diseases related to other systems of the body (Figure 1).

### 4.1. Key Mechanisms and Signaling Pathways Associated with Ferroptosis

Ferroptosis depends on the free iron content in cells, and it is different from apoptosis [43], necrosis [44], autophagy [45], and pyroptosis [46] in morphological, biochemical, and genetic aspects (Table 1). In fact, ferroptosis involves complex and diverse biochemical processes, such as the metabolism of iron, amino acids, and polyunsaturated fatty acids (PUFAs) as well as the biosynthesis of glutathione, phospholipids, nicotinamide adenine dinucleotide phosphate (NADPH), and coenzyme Q10 (CoQ_10_) [47] (Table 2).

### 4.2. The Key Role of the System x_c_^−^-GSH-GPX4 Axis

**Figure 2:**
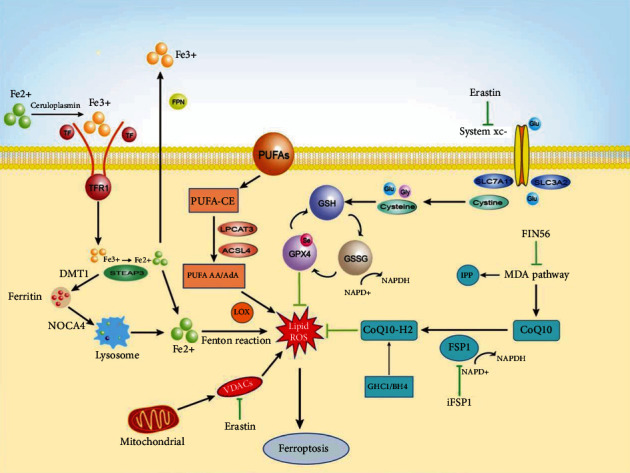
Major signaling pathways and key regulators of ferroptosis. The major regulatory pathways can be roughly divided into three categories, namely, the system x_c_^−^-glutathione-glutathione peroxidase 4 (system x_c_^−^-GSH-GPX4) pathway, iron metabolism, and the pathways related to lipid metabolism. Moreover, the ferroptosis inhibitory protein 1-coenzyme Q10-nicotinamide adenine dinucleotide phosphate (FSP1-CoQ_10_-NAD(P)H) pathway and GTP cyclohydrase 1-tetrahydrobiopterin (GCH1-BH4) axis are newly discovered independent systems that function parallel to the system x_c_^−^-GSH-GPX4 axis and can inhibit phospholipid peroxidation and ferroptosis.

System x_c_^−^, an antiporter composed of the catalytic subunit solute carrier family 7 member 11 (SLC7A11) and the chaperone subunit solute carrier family 3 member 2 (SLC3A2), can simultaneously transfer glutamate out of the cell and cystine into cells [48]. A previous study has reported that interferon-*γ* (IFN*γ*), a glycosylated protein that functions in tumor rejection, can downregulate the levels of SLC7A11 and SLC3A2, increase the ROS levels, and decrease the mitochondrial membrane potential in hepatocellular carcinoma cells. In fact, these changes are accompanied by a decrease in the system x_c_^−^ activity, which, in turn, sensitizes the cells to ferroptosis [49]. For example, P53 can inhibit system x_c_^−^ uptake of cystine by downregulating the expression of SLC7A11, thereby affecting the activity of GPX4, leading to decreased cellular antioxidant capacity, accumulation of lipid ROS, and ultimately resulting in ferroptosis [50, 51]. Additionally, system x_c_^−^ is upregulated during ischemic stroke, thereby aggravating glutamate excitotoxicity after cerebral ischemia, and this can be effectively suppressed by the action of ferroptosis inhibitor Ferrostatin 1 (Fer-1) [14]. Cystine, which is transferred into cells by system x_c_^−^, is necessary for the synthesis of glutathione (GSH), a peptide capable of scavenging oxidative free radicals of phospholipids on cell membranes. GSH also decreases ROS under the action of glutathione peroxidases (GPXs). Inhibiting the activity of system x_c_^−^ affects the synthesis of GSH by inhibiting the absorption of cystine, which results in a decrease in GPXs activity, accumulation of lipid ROS, cell antioxidant capacity, and ultimately the occurrence of ferroptosis and oxidative damage [52, 53]. Additionally, free Fe^2+^ can oxidize lipid peroxides-OH (L-OOH) to produce highly reactive phospholipids-H (L-O). These free radicals can damage PUFAs through chain reactions, ultimately causing extensive membrane and mitochondrial damage as well as inducing ferroptosis [54]. GSH can be used as a binding ligand for free iron, thereby preventing it from reacting with H_2_O_2_ to generate highly cytotoxic hydroxyl free radicals [55] (Figure 2).

Among the GPX family, GPX4 is the key enzyme that limits the accumulation of lipid peroxides and consequently protects the cells from lipid hydroperoxides. A recent study has identified a novel upstream regulatory mechanism of GPX4, in which the activation of mammalian target of rapamycin complex 1 (mTORC1) leads to the SLC7A11-mediated cystine uptake, which, in turn, mediates the GPX4 biosynthesis [56]. Inhibiting the effects of GPX4 can cause L-OOH to produce large amounts of alkyloxy radicals, which cause further membrane and mitochondrial damage [57]. Furthermore, GPX4 can consume two molecules of GSH to reduce L-OOH to L-OH, and in the process, GSH is oxidized to oxidized glutathione (GSSG). Thereafter, the GSSG is reduced to GSH by NADPH-dependent reductase, and it enters the next cycle [57]. On the one hand, inhibitors such as erastin can indirectly reduce the synthesis of GSH, and lack of GSH will affect the activity of GPX4 [58]. On the other hand, compounds such as RSL3 can directly acts on GPX4 and inactivates it thereby reducing the accumulating ROS and antioxidant capacity further resulting in ferroptosis [51]. Through the discovery of the system x_c_^−^-GSH-GPX4 axis, it has witnessed rapid progress in better understanding of ferroptosis. Zhang et al. revealed that ferroptosis inhibitor SRS16-86 could reduce the ferroptosis-related metabolite 4-hydroxylnonenal (4HNE) and upregulated GPX4 and GSH, reducing the redox damage in SCI rats [59]. The antigliotic effect of deferoxamine (DFO) is also consistent with its effect on GPX4 upregulation [60], which might have an impact on the system x_c_^−^-GSH-GPX4 pathway in inhibiting ferroptosis. Another study showed that liproxstatin-1 (Lipro-1) not only inhibits mitochondrial lipid peroxidation but also restores the expression of GSH, GPX4, and ferroptosis inhibitory protein 1 (FSP1). In fact, the Lipro-1-induced restoration of GPX4 to its normal levels ensures inhibition of the ferroptosis signal within the nucleus, while the increased GSH levels enhance the antiferroptosis system [61]. In conclusion, the system x_c_^−^-GSH-GPX4 axis is the mainstay of ferroptosis. It is possible to alleviate the pathological progress of PNI, promote nerve cells regeneration and functional recovery by regulating the system x_c_^−^-GSH-GPX4 axis after PNI.

### 4.3. Lipid Peroxidation

Since PUFAs, especially arachidonoyl (AA) and adrenoyl (AdA), are extremely sensitive to oxidation, they form the main substrates of lipid peroxidation in ferroptosis; particularly, phosphatidylethanolamine (PE), which contains AA, is the key phospholipid that induces ferroptosis in cells [62]. Incidentally, the PUFAs on cell membranes are oxidized by LOX to form L-OOH. Lipidomic analysis also showed that AA and adrenoic acid containing phophatidyl ethanolamine are lipid products of ferroptosis and can spontaneously peroxidation in the presence of hydroxyl radical produced by the Fenton reaction [63]. When a large amount of Fe^2+^ accumulates in the cytoplasm, lipid hydrogen peroxide can form toxic lipid ROS, leading to cell damage. Such lipid free radicals will capture electrons near PUFAs and initiate a new round of lipid oxidation, causing more sever oxidative damage [64]. Moreover, under the Fenton reaction, lipid peroxides are converted to LO-, and the PUFAs on the membrane are broken, thereby triggering ferroptosis [42]. Incidentally, lysophosphatidylcholine acyltransferase 3 (LPCAT3) and acyl-CoA synthetase long-chain family member 4 (ACSL4) are involved in the activate PUFAs, biosynthesis of PE, and affect the transmembrane properties of PUFAs [65]; both of which serve as important molecular markers of ferroptosis. Guo et al. reported that overexpression of ACSL4 is accompanied by the inactivation of GPX4 in chronic constriction injury (CCI) rats; this implies that upon GPX4 inhibition during CCI-induced ferroptosis, the lipid oxidation process may depend on the activation of ACSL4 [66]. Therefore, downregulating the expression of ACSL4 and LPCAT3 may reduce the accumulation of lipid peroxide substrates and inhibit ferroptosis. As mentioned above, it is important to understand these signaling molecules and their transduction pathways in the pathophysiology of ferroptosis in the process of PNI.

### 4.4. Reactive Oxygen Species

Many cellular processes generate ROS and induce intracellular oxidative stress, thereby leading to tissue and cell damage that can result in NP [67]. Moreover, oxidative stress can cause damage to mitochondria, which further increases the oxidative stress and promotes the development of NP [68]. When the concentration of ROS exceeds the normal physiological range and the homeostasis is disturbed, it can lead to cytotoxicity; hence, low concentrations of ROS should be released to the extracellular environment to protect the nerve cells from the damage caused by these molecular signaling pathways [69]. Excessive iron is capable of reacting with H_2_O_2_ or HO^−^ radicals; in fact, Fe^2+^ is conducive to the production of ROS, and it promotes lipid peroxidation, thereby inducing ferroptosis [41]. Bowen et al. demonstrated that an increase in the cytochrome oxidase subunit 4 isoform 2 (Cox4i2) expression, which increases cytochrome oxidase (COX) activity, can promote ROS production, thereby leading to ferroptosis in human herpes virus 7- (HHV7-) infected SCs; additionally, the depletion/knock-down of Cox4i2 can suppress HHV7-induced ferroptosis and apoptosis of SCs [30]. Another study has revealed that post-PNI, several proteins exhibit cellular responses to the increased levels of ROS and oxidative stress; moreover, redox-dependent metabolic processes are upregulated, indicating their involvement in the development of NP [70]. As indicated by another study, S100A4, a small calcium-binding protein, is highly upregulated after PNI, and mice lacking S100A4 exhibit increased mechanical hypersensitivity after PNI. This result indicated that downregulating S100A4 may result in an increased NP hypersensitivity [71]. In conclusion, a proper control of the ROS levels can reduce the occurrence of ferroptosis and help to alleviate the symptoms of PNI.

### 4.5. Mitochondrial Dysfunction

Mitochondria, which is the main regulators of oxidative phosphorylation, plays a key role in oxidative stress. One of the most important functions of the mitochondria is to provide energy, necessary for cellular metabolism, via oxidative phosphorylation. Mitochondria form the major organellar site for iron, amino acid, fatty acid, and carbon metabolism [72]. Iron ions can reach the mitochondrial matrix through the outer and inner mitochondrial membrane, thereby regulating the physiological functions of important organelles in mitochondria [73]. Moreover, mitochondrial ROS are important factors for inducing apoptosis as well as ferroptosis. Mitochondria are also involved in regulating iron homeostasis in the nervous system, and recent studies of the nervous system have reported that the occurrence of ferroptosis in neurodegenerative diseases is closely related to mitochondria [74]; studies have confirmed that mitochondria play a role in promoting the death signal imposed by increased lipid peroxidation in neuronal cells [75, 76]. Therefore, mitochondria may regulate ferroptosis from multiple links, thereby affecting the progression of neurodegenerative diseases. Mitochondria provide specific lipid precursors required for ferroptosis through fatty acid metabolism and glutamine hydrolysis. In addition, mitochondrial lipid peroxidation in vitro can cause mitochondrial lipid peroxidation and mitochondrial damage through the diffusion of oxidative stress, thereby disrupting mitochondrial regulation of iron homeostasis and ultimately leading to ferroptosis. Abdalkader et al. have reported that the Nrf2 signaling pathway is involved in regulating mitochondrial function and affects many molecular aspects of ferroptosis, suggesting treatment against Nrf2 exert an antiferrous attenuating effect in cancer cells and has a beneficial influence on many neurodegeneration models [75]. Interestingly, mitoquinone (MitoQ), a mitochondria-specific ROS scavenger, can protect the integrity and function of mitochondria, thereby making it an effective strategy for preventing ferroptosis in cells. This cytoprotective effect of MitoQ is associated with the selective attenuation of mitochondrial ROS formation [77]. Additionally, the VDACs, which are abundantly located in the outer mitochondrial membrane, transport ions and metabolites; this allows them to control the crosstalk between mitochondria and cells during oxidative stress, which, in turn, plays an essential regulatory role in ferroptosis [78]. In 2007, Yagoda et al. reported that erastin is an activator of VDAC2 and VDAC3, and hence, it can cause an increase in the mitochondrial transmembrane potential, causing mitochondrial dysfunction and leading to large amounts of released oxides and further inducing ferroptosis [79]. In addition, a subsequent study revealed that VDAC2 is the target of carbonylation during RSL-3-induced ferroptosis [80]. These results also suggest that VDAC plays a role in regulating mitochondrial damage during ferroptosis (Figure 2).

### 4.6. Mevalonate Pathway

The mevalonate (MVA) pathway, one of the most important pathways of cellular metabolism, generates isopentenyl pyrophosphate (IPP), CoQ_10_, and cholesterol; all of which have an impact on ferroptosis at different levels [81]. The MVA pathway is vital for the maturation of GPX4. Interestingly, FIN56 can block the synthesis of GPX4 and hence reduce its expression. Moreover, it can degrade GPX4 as well as deplete the endogenous antioxidant CoQ_10_. Hence, FIN56 is one of the inducers of ferroptosis [82]. Therefore, simvastatin-induced downregulation of the rate-limiting enzyme of the MVA pathway will impair the effective translation of GPX4 and make the cells sensitive to ferroptosis [83]. A recent study has reported that the expression of the mitochondrial apoptosis inducing factor 2 (AIFM2) can compensate for the function of GPX4 in the human GPX4 deletion cancer cells, and it has been renamed as the FSP1 since it can inhibit ferroptosis without the involvement of GPX4 [84]. To eliminate lipid peroxidation, first, CoQ_10_ needs to undergo FSP1-mediated conversion into the lipophilic antioxidant CoQ_10_-H_2_, which can subsequently remove PLOOH, terminate the antilipid oxidation chain reaction, and finally inhibit ferroptosis. The FSP1-CoQ_10_-NAD(P)H pathway is considered to be an independent ferroptosis-inhibitory pathway, which exists as a parallel system and cooperates with the x_c_^−^-GSH-GPX4 pathway [84, 85]. The expression of FSP1 was closely associated with ferroptosis resistance in tumor cells, for example, FSP1 mediates ferroptosis resistance in lung cancer cells in culture and mouse tumor xenografts [86]. These findings suggested the effect of NAD(P)H in the MVA pathway through the loss of ubiquinone convergence on FSP1 and thereby predicted sensitivity to ferroptosis. Additionally, another GPX4-independent ferroptosis-blocking pathway, involving the GTP cyclohydrolase 1 gene (*GCH1*), has been identified as the rate-limiting step in the production of the metabolite tetrahydrobiopterin (BH4) [87]. The BH4 can inhibit ferroptosis by aiding the formation of reduced CoQ10 and by blocking the peroxidation of specific lipids [88]. Therefore, the GCH1-BH4 axis is another independent parallel system that cooperates with the system x_c_^−^-GSH-GPX4 pathway (Figure 2).

### 4.7. Ferroptosis and Ferritinophagy

Ferritinophagy is a selective form of autophagy that induces ferroptosis through the degradation of ferritin and triggers labile iron overload, lipid peroxidation, membrane damage, and cell death [89]. Under normal physiological conditions, ferritinophagy maintains the iron balance within the cells. However, hyperactivation of ferritinophagy may lead to excessive iron deposition, which, in turn, the subsequent increase of iron ions in LIP induces lipid peroxidation through the Fenton reaction, thereby leading to the structural breakdown and rupture of the cell membrane and ultimately inducing ferroptosis [90, 91]. Initially, Mancias et al. reported that the nuclear receptor coactivator 4 (NCOA4) is enriched in autophagosomes [92]. Thereafter, studies have revealed that ferritinophagy is mediated by NCOA4, which transports intracellular ferritin to autophagy lysosomes for degradation, followed by the release of free iron ions. These results confirmed NCOA4 as a selective cargo receptor for ferritinophagy; incidentally, it plays an important role in maintaining iron homeostasis and participates in the iron-dependent physiological processes, and it has been reported that the sensitivity of ferroptosis is affected by NCOA4 levels. A reduction in the NCOA4 levels can decrease the ferritinophagy flux and further reduce the sensitivity to ferroptosis, while its overexpression can increase the LIP to promote the accumulation of ROS and induce the occurrence of ferroptosis [93].

### 4.8. Ferroptosis in the Schwann Cells after Peripheral Nerve Injury

Oxidative stress enhances the intracellular levels of ROS and subsequently causes DNA damage, thereby leading to further nerve injury. Aggregation of ROS, which has a strong oxidizing ability especially for lipids and DNA and often causes lipid peroxidation, leads to a high degree of neuronal or SCs degeneration. Moreover, iron ions can also lead to SCs damaged by producing ROS. During the Wallerian degeneration, SCs lose their characteristic of myelinating axons and shift into the state of developmental promyelinating cells. In the meantime, SCs dynamics during the Wallerian degeneration is related to oxidative stress. Heme oxygenases (HOs) are involved in the oxidative degradation of heme into ferrous iron, carbon monoxide, or biliverdin/bilirubin. Overproduction of ferrous iron by HOs increases ROS, which have deleterious effects on living cells [13]. Hence, the key molecule for understanding the exact mechanism of Wallerian degeneration in the PNS is closely related to oxidative stress-mediated HOs in SCs. By the way, HOs are mediators of oxidative injury and affect cellular redox homeostasis, which indicates that HOs are related to the occurrence of ferroptosis. Previous studies have reported that DNA damage exists, irrespective of the acute or chronic phases of nerve injury, thereby causing a series of adverse reactions, such as intracellular protein oxidation and inactivation [94, 95]. Apart from DNA and protein damage, lipid metabolism also plays a key role in the neuroimmune communication during nerve injury-induced NP. Several lipid receptors have been identified as important mediators of the onset, maintenance, and resolution of NP [96]. These various intracellular metabolism disorders also lead to ferroptosis in SCs, further exacerbating the process of nerve injury. Study has reported that inhibiting the process of ferroptosis can improve the pain threshold of rats in the pathological pain model, and both ferrostatin-1 and the iron chelator could reduce neuronal degeneration in animal models [66]. Moreover, inhibition of the ferroptosis pathway has successfully alleviated oxidative nerve injury as well as mechanical or thermal hypersensitivities, thereby revealing that ferroptosis occurs in the SCs of injured tissues after PNI [30, 97].

### 4.9. Ferroptosis May Occur in Macrophages after Peripheral Nerve Injury

During the Wallerian degeneration, activation of SCs is accompanied by recruitment of immune cells into the lesion site such as lymphocytes, macrophages and neutrophils [98]. Among these different cells, macrophages not only play a key role in promoting SCs activation and removing myelin debris but also contributing to the creation of permissive environment for axonal regeneration by releasing different proregenerative factors such as growth factors, chemokines, cytokines, and extracellular matrix (ECM) molecules [99]. In addition, macrophages-derived endosomes also play a crucial role in the Wallerian degeneration and axonal regeneration [100]. One of the sources of ROS in macrophages is *γ*-glutamyltransferase (GGT), which is associated with exposure to neuroinflammation. Oxidative injury and clinical signs of neuroinflammation can be suppressed by targeting GGT activity [101]. Proper ROS levels help immune protection; however, excessive production of ROS will damage the cell membrane and DNA, leading to cell death [102]. Ferroptosis is a kind of RCD caused by high ROS levels, indicating the relationship between ferroptosis and macrophages. After PNI, local iron ions accumulate in macrophages and are accompanied by the generation of lipid peroxidation and the release of ROS by the Fenton reaction, resulting in ferroptosis of macrophages. Iron accumulation in macrophages can affect other cells in different tissues [102]. In SCs, once the balance is broken by the accumulation of iron in macrophages, the unregulated iron export will also lead to systematic iron overload, which further creates chances for the occurrence of ferroptosis in PNS. Therefore, the association between macrophages and ferroptosis is closely related to the progression of PNI.

## 5. Ferroptosis and Peripheral Nerve Injury

**Figure 3:**
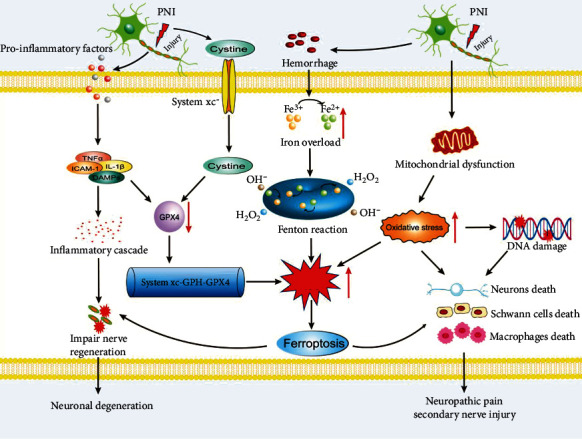
Overview of the role of ferroptosis in peripheral nerve injury. After PNI, there is a local iron ions overload, generation of large amounts of reactive oxygen species (ROS), and an increased excitatory toxicity of glutamate. Additionally, mitochondrial shrinkage and outer membrane ruptures lead to mitochondrial dysfunction and DNA damage, thereby increasing oxidative stress and enhancing the ROS levels. This further induces nerve cell death as well as ferroptosis, such as neuron death, the Schwann cell death, and macrophage death, ultimately causing secondary nerve injury. Furthermore, the accumulation of various inflammatory factors might downregulate glutathione peroxidase 4 expression and aggravate neuronal degeneration or tissue damage, leading to ferroptosis and neuropathic pain.

In recent years, the relationship between iron and PNI has attracted increasing attention. In normal conditions, the system xc^−^-GSH-GPX4 axis is responsible for the extracellular transport of cystine and the intracellular transport of glutamat [103]. A microenvironment is established upon nerve injury, which consists of axons, SCs, blood vessels, inflammatory factors and cells, extracellular matrix, and connective tissue [104]. The bleeding in the local environment promotes iron ions overloaded, increasing ROS levels of the Fenton reaction and participating in ferroptosis [105]. The enhanced expression of cystine in the extracellular matrix might also take part in the GPX4 regulation and lipid peroxidation [106, 107]. In addition, the inflammatory microenvironment releases proinflammatory factors, resulting in an inflammatory cascade, which reduces GPX4 expression and increases lipid peroxidation through the system x_c_^−^-GSH-GPX4 axis, further leading to ferroptosis [108]. Taken together, ferroptosis is activated after nerve injury with increasing evidences of morphology, biochemistry, and biomolecules reported in the papers. The ferroptosis causes the further damage of SCs and axons, which exacerbates the progression of nerve damage and leads to secondary nerve injury, NP, or neuroinflammation (Figure 3).

### 5.1. Local Iron Overload and Ferroptosis Occur after PNI

Iron is an essential cofactor for metabolic processes, which is required for axonal function and regeneration. Due to the close proximity to axons, SCs are a likely source of iron for axonal in the PNS [109]. SCs express the molecular machinery to release the iron exporter, such as FPN and the ferroxidase ceruloplasmin (CP), and accumulate iron as FPN requires to partner with CP to export iron. SCs and axons also express the iron importer TFR1, revealing their ability for iron uptake. What is more, TFR1 and FPN are localized at the nodes of the Ranvier and Schmidt-Lanterman incisures so that theses axonal sites are in close contact with SCs cytoplasm [23]. The subcellular distribution of ferritin can be observed with a high degree of accuracy using extremely sensitive detection techniques, such as magnetic force microscopy (MFM) or transmission electron microscopy; in fact, this has helped to confirm the increased aggregation of ferritin in injured nerve tissues [110]. Excess iron is toxic to nerve cells, such as SCs and neurons, because the iron overload can promote lipid peroxidation through the Fenton reaction as well as ROS production, which, in turn can mediate ferroptosis. Wang et al. revealed that ferroptosis is closely related to the neuronal reduction of glial cell-activated iron ion accumulation, thereby highlighting the role of iron in NP [39]. In another related study, after adding ferrous ions to the culture dish of nerve cells, it was observed that large amounts of lipid peroxidation metabolites was proportional to the level of iron and positively correlated with neuronal inactivation [111].

In PNS, studies have confirmed that loss of lipid receptor G2A can alleviate mechanical hypersensitivity in case of acute, nerve injury-induced peripheral NP [96, 112]. This may occur due to changes in the neuroimmune response, specifically the reduced immune cell infiltration of the injured nerve. Gao et al. found a decrease in SCs viability along with a decrease in GSH content and an increase in MDA, ROS, and Fe^2+^ contents during erastin-induced SCs. In the meantime, it was found that the Nrf2/HO-1 pathway was activated, which is considered one of the most important key pathways of ferroptosis. Nrf2/HO-1 pathway was inhibited by c-Jun overexpression and exerts an antiferroptosis effect [113]. C-Jun is a key regulator of the response of SCs to PNI, which is low or absent in normal condition but rises rapidly after nerve injury. Inhibiting ferroptosis in SCs via overexpression of c-Jun may contribute to the recovery of peripheral nerve function. These results also provide a molecular basis for understanding the role of ferroptosis during PNI. In the CCI-induced NP rat models, GPX4 expression was downregulated, and ACSL4 expression was enhanced, suggesting the participation of ferroptosis in the CCI [39]. Furthermore, ferroptosis inhibitor, Fer-1, could relieve the pain and hyperalgesia responses by increasing GPX4 expression [39]. These results also suggest the existence of ferroptosis during PNI.

### 5.2. Mitochondrial Damage after PNI

In the acute phase of nerve tissue injury, transmission electron microscopy can help us to observe mitochondrial shrinkage, outer membrane rupture, and cristae disappearance; all of which are characteristic morphological changes occurring in the mitochondria during ferroptosis [114]. Study has showed that lack of iron export from SCs reduces mitochondrial iron in axons as detected by reduction in mitochondrial ferritin, affects localization of axonal mitochondria at the nodes of the Ranvier and Schmidt-Lanterman incisures, and impairs axonal regeneration following sciatic nerve injury [23]. When mitochondria are damaged, depletion of intracellular GSH and production of ROS increases, and the intracellular oxidative stress levels increase subsequently. Oxidative stress activates intracellular signaling pathways mediated by cytokines and growth factors, further leading to cell and tissue damage and resulting in NP [114, 115]. In the meantime, oxidative stress also causes changes in mitochondrial structure and function, exacerbating oxidative stress and promoting the process of NP [115]. The nervous system has a high demand for energy, and mitochondria are the central organelles that produce energy, thus, once mitochondria are damaged, neuronal damage can be induced, leading to NP [39]. PNI induces aberrant changes in mitochondrial morphology, characterized by increase in the mitochondrial membrane density and decrease in the mitochondrial length [66]. However, intervention with ferroptosis inhibitors leads to a gradual restoration of the mitochondrial morphology to its normal shape, hence confirming the involvement of ferroptosis in CCI and NP [39, 67]. On the contrary, the inhibition of VDACs can cause mitochondrial dysfunction and release large amounts of oxides, further inducing ferroptosis [116]. These findings suggest that SCs contribute to the delivery of iron to axonal mitochondria, required for peripheral nerve repair. In conclusion, strategies to improve mitochondrial function have successfully prevented and reversed NP in preclinical animal models [117]. Therefore, inhibiting ferroptosis and protecting mitochondrial function may play an important role in treatment of PNI.

### 5.3. Ferroptosis and Neuroinflammation in PNI

Neuroinflammation, a localized inflammation occurring in the nervous system in response to trauma, neurodegeneration, or autoimmunity, is a complex process of interaction between glial cells and the surrounding immune cells of the nervous system [118]. To investigate the possible functional consequences of neuronal inflammasome, Molnar et al. evaluated sciatic nerve regeneration in animal models, revealing that PNI initiates an inflammatory process both within injured axons or peripheral nerve. This process includes NLRP3 inflammasome in motoneurons and subsequently release of the proinflammatory cytokine IL-1*β*, resulting in impaired regeneration in peripheral components of the affected motoneurons [119]. In addition to this, activation of glial cells and peripheral blood lymphocytes leads to the ATP-mediated release of inflammatory factors, resulting in damage to the nervous system. PNI may also induce the overproduction of multiple proinflammatory compounds and dysregulation of pain-related genes, which may act as triggers for the maintenance and emergence of neuroinflammation [120]. Nerve injury-induced NP is characterized by a strong inflammatory component that involves immune cell migration to the injured site of the peripheral nerve [112]. When oxidative stress is maintained for a long time, macrophages accumulate massive amounts of oxidized lipids and proteins, resulting in the occurrence of ferroptosis. This is particularly relevant to PNI. Under the inflammatory microenvironment, glial cells undergo different phenotypic transformations and regulate inflammation via cellular feedback and communication mechanisms, ultimately causing the proinflammatory reactions associated with neuroinflammation [121]. It has been reported that P53 can induce the occurrence of ferroptosis by enhancing the expression of spermine N1-acetyltransferase 1 (SAT1), an important rate-limiting enzyme in polyamine catabolism; the expression of which is closely related with varieties of pathological conditions including nerve injury and neurological disorders [121]. Oxidative stress and inflammatory stimuli can increase SAT1 activity, and SAT1 activation thereby promoting lipid peroxidation induced by ROS, further leading to ferroptosis and induce inflammatory injury of the nervous system [121, 122]. Moreover, studies have demonstrated that ferroptosis contributes to the pathological process of neuroinflammation, and ferroptosis inhibitors can inhibit the astrocyte activation, alleviating the process of nerve injury [121]. During nerve injury caused by ferroptosis, the release of endogenous and exogenous pathogens or cellular damage products stimulates the Toll-like receptor (TLR) family signals in glial cells, ultimately leading to neuronal damage. Additionally, GPX4 is involved in the inflammatory response and is associated with microgliosis and astrocyte activation [53]. It can also reduce the expression of inflammatory factors, namely, intercellular cell adhesion molecule-1 (ICAM-1) and tumor necrosis factor (TNF*α*) [59, 123], similar to its role in reducing the expression levels of TNF*α* and interleukin (IL)-1*β* to alleviate neuronal damage [124]. Neuroinflammation needs to be controlled to prevent secondary nerve damage during the regeneration process post-PNI. In the sciatic nerve injury mouse model, the nerve damage group exhibited a high number of proinflammatory macrophages; however, CryAB, a small heat shock protein with numerous protective functions, demonstrated an immunosuppressive effect on the cytokine secretion from these proinflammatory macrophages, thereby suggesting that it may have the potential of inhibiting proinflammatory responses after PNI [125]. The danger signal proinflammatory damage-associated molecular patterns (DAMPs) are endogenous molecules encoded directly by endogenous genes of the host, which can be released under tissue damage without the need for protein synthesis. DAMPs from proinflammatory cytokines also provide prefeedback signals that reinforce the programmed death of cells [108]. Ferroptosis often releases a large amount of DAMPs and other inflammatory factors, which, in turn, activate inflammatory cells and form an inflammatory cascade, promoting a series of inflammatory factors which contribute to peripheral nerve damage and neurological disorders [108]. Ferroptosis inhibitors can also reduce the concentration of proinflammatory factors and promote tissue repair, thereby significantly promoting the functional recovery of rats in the NP model. Therefore, in-depth research on the relationship between ferroptosis and neuroinflammation is of great significance for the treatment of PNI.

## 6. Existing Problems

Continuous studies have identified new cell death pathways associated with PNI as well as new potential therapeutic targets for the treatment of PNI. Nevertheless, antiferroptosis treatment strategies for PNI are still being explored at the cellular and animal levels, i.e., they have not been tested in clinical trials. Currently, the knowledge regarding the mechanism of ferroptosis and the relationship between ferroptosis and PNI is limited, and the existing results are not enough to progress to human studies. Therefore, more evidence is needed to verify the currently available knowledge in both *in vivo* and *in vitro* experiments. Most importantly, different RCD pathways may crosstalk, for instance, there is a complex molecular signaling that occurs between autophagy and apoptosis [126]; however, it is unclear whether ferroptosis communicates with other cell death pathways during PNI. Wu et al. reported that autophagy can increase the intracellular free iron concentration by degrading ferritin in neurons; this, in turn, promotes ferroptosis, thereby revealing the close connection between autophagy and ferroptosis in RCD [127]. Therefore, further investigations are necessary to determine whether there are other cross-communications between ferroptosis and the different cell death pathways.

## 7. Conclusions

Ferroptosis, a newly discovered form of cell death, has attracted the attention of many scholars, and several studies have been conducted to elucidate its role in PNI. In this review, we elucidated the mechanism of ferroptosis with respect to iron overload, system x_c_^−^-GSH-GPX4 pathway, ROS accumulation, lipid peroxidation, mitochondrial dysfunction, and their effects on PNI. Increasing evidence has demonstrated that nerve injury is closely associated with abnormal iron metabolism that can cause ferroptosis, and inhibiting ferroptosis can alleviate tissue damage as well as promote functional recovery after PNI. In conclusion, ferroptosis plays an essential role in PNI, and extensive research is necessary to improve the theoretical knowledge base as well as develop promising treatment strategies for PNI.

## Figures and Tables

**Table 1:** The features of different kinds of cell death pathways.

	Apoptosis	Autophagy	Necroptosis	Ferroptosis	Pyroptosis
Morphological features	Apoptotic bodies formation, normal mitochondrial structure	Autophagosome formation, mild dilatation of mitochondrial structure	Dilatation of nuclear membrane, cytoplasm, and mitochondria swelling	Mitochondrial membrane rupture, normal nucleus	Cytoplasmic membrane ruptured, pyrosomes formation, and inflammasome formation
Biochemical features	DNA fragmentation	Lysosomal activity	Depletion of ATP	Iron overload, lipid peroxidation	Caspase-1 activation, gasdermin cracking
Common regulatory pathways	Caspase activation, cleavage of caspase substrates, Bcl-2, and MMP family	ATG family, cleavage of p62, and LC3 conversion	Damps release, TNF-R1, and RIP1/RIP3-MLKL pathway,	System xc-GSH-GPX4 pathway, FSP1-CoQ10-NAD(P)H pathway, and GCH1-BH4 axis	Caspase-1-IL-1*β*-Gasdermin D, NLR, and AIM2 family;
Key genes	Bcl-2, caspase, p53, and MMP-2	ATG5, LC3, and Beclin-1	RIP1, RIP3	GPX4, ACSL4, FSP1, SLC7A11, and NCOA4	Caspase-1, Caspase4/5/11, GSDMD, IL-1*β*, IL-18, and NLPR3

**Table 2:** The major regulatory mechanisms and common biomolecules/compounds associated with ferroptosis.

Influence factors	Compounds or drugs	Mechanism
Iron	DFO	Inhibit accumulation of iron
	SRS 16-68	Upregulate GPX4, GSH
	NCOA4	Degrade and release iron
System x_c_^−^	Erastin	Inhibit system x_c_^−^
	Glutamate	Prevent cystine import
Lipid peroxidation	Fer-1	Reduce iron deposition
	Lipro-1	Decrease MDA levels
	iFSP1	Block the effects of FSP1
	BH4	Block lipid peroxidation
GPX4	RSL-3	Inhibit GPX4
	FIN56	Deplete antioxidant CoQ_10_ and degrade GPX4
ROS	Antioxidant	Terminate antilipid oxidation chain reaction
